# An ensemble reconstruction of global monthly sea surface temperature and sea ice concentration 1000–1849

**DOI:** 10.1038/s41597-021-01043-1

**Published:** 2021-10-04

**Authors:** Eric Samakinwa, Veronika Valler, Ralf Hand, Raphael Neukom, Juan José Gómez-Navarro, John Kennedy, Nick A. Rayner, Stefan Brönnimann

**Affiliations:** 1 Oeschger Center for Climate Change Research, University of Bern, Bern, Switzerland; 2 Institute of Geography, University of Bern, Bern, Switzerland; 3 Department of Geography, University of Zurich, Zurich, Switzerland; 4 Department of Geosciences, University of Fribourg, Fribourg, Switzerland; 5 Department of Physics, University of Murcia, Murcia, Spain; 6 Met Office, Exeter, United Kingdom

**Keywords:** Palaeoclimate, Physical oceanography

## Abstract

This paper describes a global monthly gridded Sea Surface Temperature (SST) and Sea Ice Concentration (SIC) dataset for the period 1000–1849, which can be used as boundary conditions for atmospheric model simulations. The reconstruction is based on existing coarse-resolution annual temperature ensemble reconstructions, which are then augmented with intra-annual and sub-grid scale variability. The intra-annual component of HadISST.2.0 and oceanic indices estimated from the reconstructed annual mean are used to develop grid-based linear regressions in a monthly stratified approach. Similarly, we reconstruct SIC using analog resampling of HadISST.2.0 SIC (1941–2000), for both hemispheres. Analogs are pooled in four seasons, comprising of 3-months each. The best analogs are selected based on the correlation between each member of the reconstructed SST and its target. For the period 1780 to 1849, We assimilate historical observations of SST and night-time marine air temperature from the ICOADS dataset into our reconstruction using an offline Ensemble Kalman Filter approach. The resulting dataset is physically consistent with information from models, proxies, and observations.

## Abstract (summary)

**Table:** 

Measurement(s)	temperature of sea surface • sea ice concentration
Technology Type(s)	computational modeling technique
Sample Characteristic - Environment	ocean

Machine-accessible metadata file describing the reported data: 10.6084/m9.figshare.16592657

## Background & Summary

The oceans cover approximately 71% of the Earth’s surface and have a significant influence on atmospheric processes by supplying heat and moisture, hence driving the atmospheric circulation, from micro to macro scale. Forced Atmospheric General Circulation Models (AGCMs) setups are used in a broad variety of applications, e.g., the analysis of how Sea Surface Temperature (SST) patterns impact on the atmosphere, data assimilation appoaches, and so on. Oceanic boundary conditions such as SST and Sea Ice Concentration (SIC) drives a substantial fraction of climate variability. Various SST and SIC products have been developed to provide spatial and temporal representations of the global ocean state^1,2^ in the recent period of dense instrumental records (1850–present). However, SIC depends on climatological averages with less variability before the inception of satellite measurements. There are few of these SST products that extend its temporal coverage back beyond 1850^3–6^. One of such is the International Comprehensive Ocean-Atmosphere Data Set (ICOADS) gridded SST product. The ICOADS archive contains the world’s most comprehensive and extensive historical surface marine data collection, which extends as far back as the 16th century, with SST observations only available from 1800 onwards. However, the observations are spatially less complete particularly before the 21st century and hence cannot be used in historical AGCM simulations. An improvement based on this dataset is implemented in the Simple Ocean Data Assimilation with Sparse Input (SODAsi) framework^3^, which provides spatially complete monthly SSTs for the period 1815–2013. As the name implies, SODAsi incorporates very sparse observational inputs from ICOADS into climate model simulations, and its does not extend back beyond 1815.

Similarly, there are existing SST reconstructions which made use of climate information from various paleoclimate proxies. These proxies have been demonstrated to provide plausible and useful evidence for inferring large-scale climate variability before the beginning of the instrumental era^4,7,8^. However, the information that they can provide is limited as they are defined by two important time variables, namely resolution, and span. The latter refers to the range of time from which a given type of proxy exists, while the former is indicative of how much detail is retrievable from such an archive. Typically, proxies with high temporal resolutions, such as corals that can provide paleoclimate information on a monthly timescale, do not extend far back-in-time. On the other hand, tree rings correspond to a particular growing season within a year, and as such are annually resolved but cover a wider range of time than corals. This makes it somewhat difficult to discuss coherence and spatial patterns when combining proxies that span different time-slices, in multi-proxy climate field reconstructions. However, evolving climate reconstruction methods have taken this into account by combining proxies in a way that preserves low-frequency variability from records which can only provide useful information on decadal, centennial, or even longer timescales. There are several annually resolved temperature reconstructions. Prominent temperature reconstructions include one which investigates the origin of the Little Ice Age anomalies^4^ and the Last Millenium Reanalysis products^5^, which provides a framework for annual mean temperature reconstruction. The former has been used for AGCM simulations^9^, but with the annual fields augmented with intra-annual and sub-grid scale variability. As in many climate field reconstructions, the augmentation technique assumes the same statistical relationship between the reconstruction and available observations during calibration and reconstruction periods.

**Fig. 1:**
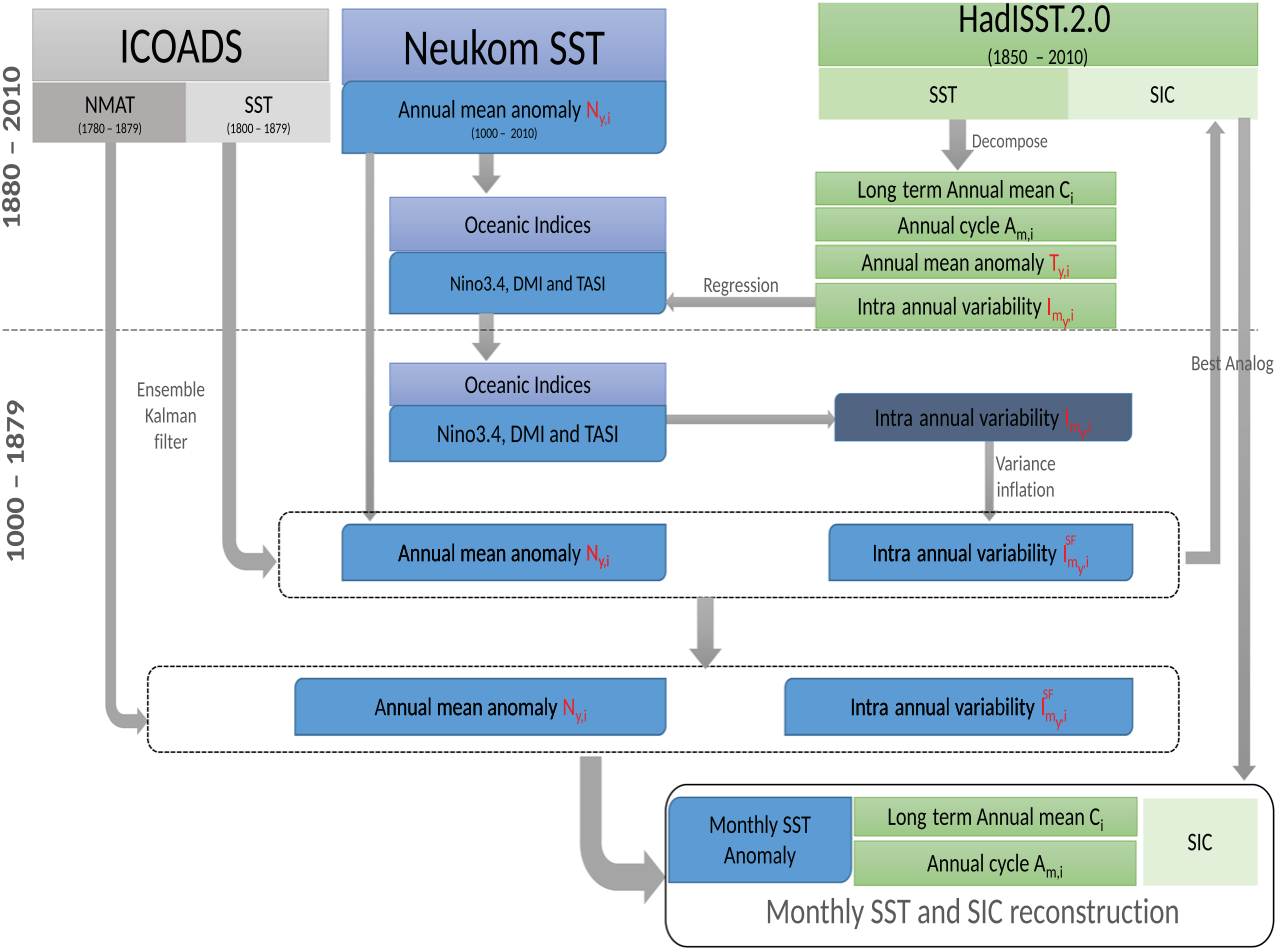
Schematic diagram, showing sequence of steps involved in reconstructing historical SST and SIC.

Our approach fills this gap, by starting from an ensemble of annual reconstructions^8^ and augmenting them with intra-annual and sub-grid scale variability from an ensemble of historical observations in a way that the annual means of the coarse resolution SST reconstructions are preserved. Furthermore, we utilize a large-body of historical observational inputs from ICOADS (1780–1849) in an offline data assimilation approach. The best sea ice analogs are selected based on a measure of similarity between subpolar and midlatitude SSTs of our reconstruction and HadISST SIC, and the resulting SST and SIC fields will enable the proper spatial and temporal representation of the historical state of the ocean, and drive the dynamics of the AGCM. A schematic of the reconstruction procedure is given in Fig. 1.

## Methods

### Data

The SST reconstruction approach depends on existing annual multi-proxy temperature reconstructions^8^, and monthly SST observations in the recent period. The annual reconstruction is augmented in an ensemble approach to sample the distribution of uncertainty in our reconstruction. In this subsection, we describe the datasets (Table 1) utilized in reconstructing SST and SIC for historical simulations in two parts, namely training datasets and historical marine observations. The former describes the different datasets used in training linear regression models to give an estimate of SSTs at every grid point of each calendar month, while the latter gives an overview of marine observations utilized to improve the regression model outputs, wherever and whenever possible.

**Table 1:** Data record information for gridded, SST, NMAT, and multi-proxy reconstructed annual temperature products.

Data and Record	Format	Resolution	Span	Source
HadISST.2.0^50^ 10-member ensemble	NetCDF	Monthly, 1° × 1°	1850–2010	Met Office^50^, United Kingdom
Annual SST reconstruction via Analog method 100-member ensemble	NetCDF	Annual, 5° × 5°	1–2012	Neukom *et al*., 2019^8^
ICOADS SST	NetCDF	Monthly, 2° × 2°	1800–2010	ICOADS^6^
ICOADS NMAT	HDF5	Sub-daily observations	1780–1879	ICOADS^6^

#### Training dataset

The reconstructed global annual temperature anomalies utilized in this study provide global coverage over the last 2000 years^8^. Furthermore, it allows for the sampling of uncertainties by using six different reconstruction methods in an ensemble approach to produce 100-members for each reconstruction method. We compared several timeslices of all the reconstructions with climatological mean from ICOADS gridded SST (1901–1930) and found a good agreement between the analog reconstructed temperature and ICOADS SST in terms of low bias and root mean square error. Here, we utilize the spatially complete annual temperature anomalies reconstructed over the tropical year (April - March) using the analog resampling method^10^ for years 1 to 2012 CE and gridded on a 5° × 5° spatial resolution. In the analog reconstructions, a total of 18,327 years were pooled from 16 simulations from the ‘past1000’ experiments, which is based on the PMIP3 setup^10^, and therefore it is already consistent with model physics. We select 50 out of 100 realizations of the analog reconstructed annual temperature anomaly fields and interpolate them to 1° × 1° grid, using the bilinear interpolation method. Our selection ensures that the ensemble spread in the coarse resolution 100-member ensemble is covered by randomly drawing 48-members in between the members with the lowest and highest global mean values. This annual temperature fields is hereafter referred to as N_*y,i*_, with subscripts y and i indicating year and grid point, respectively.

**Fig. 2:**
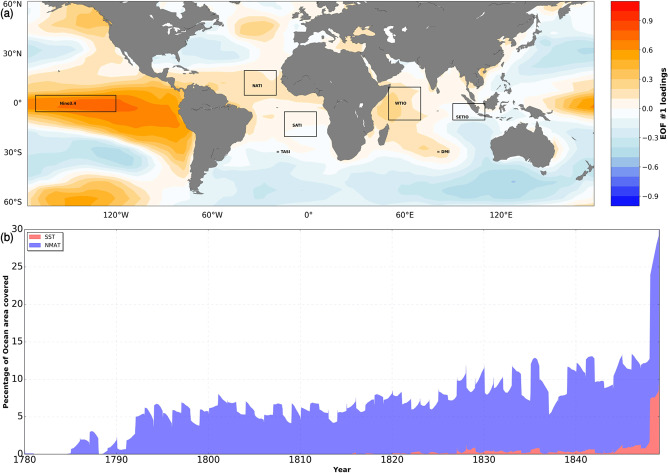
Oceanic indices and coverage of marine observations. (**a**) The spatial pattern of the first empirical orthogonal function mode (with 30.16% explained variance) of annual SST (K) reconstructed using the analog method by Neukom *et al*. (2019), calculated over 123 years (1880–2010). The polygons show regions from which we calculate climatic indices. It is important to note that extratropical variability in our reconstruction is always accounted for by tropical variability and its associated teleconnections. (**b**) Percentage of ocean area covered by marine observations (calculated on 1° × 1° grid boxes) utilized in this study, NMAT (blue) and SST (red) in the assimilation period.

For the reconstruction, climatic indices estimated from large-scale datasets can provide useful insights for inferring climate variability. Our approach aim at augmenting the annual mean reconstruction with intra-annual variability, hence we focus on oceanic modes of variability that are phase locked with the seasonal cycle. This climate-adaptive method has been applied for use in AGCM simulations^9,11^, using only El Nino Southern Oscillation (Nino3.4) as the predictor. This approach is promising and able to recover SST patterns mainly in the Pacific. We takes this into account by including all low frequency mode in the Atlantic as well as the Indian Ocean, to ensure consistency across basins. Here, we employ 3 different climatic indices, which are estimated from N_*y,i*_ for consistency. Nino3.4, which indicates the ENSO; Dipole Mode Index (DMI), which is an indicator of the Indian Ocean Dipole mode; and the Tropical Atlantic SST Index (TASI), which indicates the surface thermal conditions and meridional SST gradient between north and south tropical Atlantic^12^. While ENSO originates in the tropical Pacific, it is accompanied by teleconnection patterns in other regions and thus it is the most notable global SST indicator, leading to widespread climate anomalies^13^. ENSO exhibits seasonal phase-locking to the annual cycle, in that the peak phases are reached in boreal winter. Similar to ENSO, the Indian ocean dipole is a coupled ocean-atmosphere phenomenon usually represented by DMI^14^. DMI is used to indicate the dominant mode of variability in the Indian Ocean. Because DMI peaks during the boreal fall (September, October, November; SON) and develops in austral winter (June, July, August; JJA), information on the intra-annual scale can be gained. DMI has large influence on the climate of the tropical Indian ocean, as it is accompanied by anomalous low-level winds, and a significant contributor to tropical rainfall variability^14–16^. Lastly, TASI has been linked with the northward and southward shift of the inter-tropical convergence zone, influencing strong climate anomalies in the surrounding region^17^. Unlike Nino3.4 which is SST averaged over a certain ocean region (5° N–5° S, 170W–120W), DMI and TASI are calculated as the SST differences between two regions. While DMI is calculated as the difference between the Western Tropical Indian Ocean SST index (50° E–70° E, 10° S–10° N) and the South Eastern Tropical Indian Ocean SST Index (90° E–110° E, 10° S–0°), TASI results from the difference between North Atlantic Tropical SST Index (40° W–20° W, 5° N–20° N) and South Atlantic Tropical SST Index (15° W–5° E, 20° S–5° S) (See Fig. 2a).

To complete the training dataset, we employ a 10-member ensemble SST product from the Hadley Centre Global Sea Ice and Sea Surface Temperature (HadISST.2.0), which is an improved version of the HadISST.1.1^2^, as our Instrumental Target (IT). HadISST.2.0 is based on a blended *in-situ* and satellite data set. The *in-situ* component is a higher-resolution (1°) version of HadSST.3.1.1.0^18^ which is derived from observations in the ICOADS release 2.5^19–21^. Satellite retrievals are taken from the Along Track Scanning Radiometers (ATSR) and Advanced Very High Resolution Radiometers (AVHRR) from Pathfinder version 5^22^. The blended data set is reconstructed in a two-step process. First, Variational Bayesian Principal Component Analysis^23^ is applied to get a large-scale reconstruction based on reduced rank covariance matrix. Second, the residuals from the large-scale reconstruction are interpolated using a local optimal interpolation scheme with spatially varying hyperparameters^24^. Samples are drawn from the posterior distribution of the reconstruction at each step to generate an ensemble. IT is gridded on 1° × 1° spatial and monthly temporal resolutions, respectively. Therefore, IT and N_*y,i*_ are consistent in terms of spatial resolution but differ in terms of land sea mask. To fix this, we adapt modern-day land sea mask from IT.

#### Historical marine observations

We employ ICOADS SST version 2.5^6,20^ gridded on a spatial resolution of 2° × 2°, which is then interpolated to 1° × 1°. Although, there is a more recent release (ICOADS3.0), the dataset employed here remains unchanged in our assimilation period (1781–1879), as ICOADS3.0 only implement changes from 1960 onwards.

Marine air temperature is closely related to SST, with a global difference of less than 0.1 K between both quantities^1,25^. While disagreement between SST and marine air temperature anomalies is relatively low on the global scale, it could increase on smaller scales especially along coastlines and sea ice edges^1,26,27^. Marine air temperature from the ICOADS network are measured from ship decks and buoys and are largely affected by solar heating except at nighttime. Hence, Nighttime Marine Air Temperature (NMAT) is defined as air temperature measured from ship decks and buoys between one hour after sunset and one hour after sunrise when there is little or no effect of solar heating. Such observation exists sparsely from 1780 (Fig. 2b). We extract NMAT from ICOADS 3.0^21^ according to the above definition. This is then gridded on 1° × 1° spatial resolution to match that of the modeled SSTs.

In regions where SST observations are not sufficient to calculate monthly statistics, data voids are created resulting in less spatial coverage of the gridded data. Contrarily, we utilized all available NMAT observations, hence covering the maximum area possible. These datasets are optimally combined with our modeled SSTs, in an off-line data assimilation mode to get the best estimate of SSTs between 1781–1879.

### Reconstruction methods

In this subsection, we highlight a series of statistical techniques used in reconstructing historical SST and SIC. We describe the procedures involved in estimating grid-based linear regression coefficients by training climatic indices on IT. Since IT covers a 161-year period, we use a leave-one out type of analysis by regressing its intra-annual variability over 131 years (1880-2010) on annual climatic indices in the overlapping period, while the monthly mean SST for the overlapping 30-year period (1850–1879) is reconstructed only to validate the dataset and is not part of the released version of the reconstruction. To achieve this, we test our linear regression method in reconstructing a single-member ensemble in an alternative training and validation period. For this analysis, we regress the intra-annual variability over 129 years (1850–1978) on annual climatic indices in the overlapping period, while we used the monthly mean SST for an overlapping 30-year period in the satellite era (1980–2009) for validating the resulting dataset. The selected validation period (1850–1879) represents the time where the low resolution proxy-based annual temperature reconstruction is comparable to HadISST which uses sparse observational input prior to 1870^28^. Similarly, the proxy-based annual temperature reconstruction also uses somewhat sparse proxy inputs that are infilled to achieve global coverage. Apart from being generally time consuming, other methods like bootstrapping could undervalue extremes thereby affecting the variability of the reconstruction^29^. Furthermore, the results may vary significantly depending on the representative sample. Because the number of ensembles differs between IT (10) and N_*y,i*_ (50), we calibrate 5-members N_*y,i*_ on each member of IT. This ensures equal representation based on sample spaces of IT, across the 50 ensemble members. However, since N_*y,i*_ is already based on 50 initial sample spaces, our design here will pull the spaces closer to each other, which however will not have a significant effect on the results. We also describe the implementation of a data assimilation scheme in which historical marine observations are optimally combined with estimates of SST, obtained from regression models. Lastly, we describe the sequence of steps involved in reconstructing SIC for the same period.

#### Model formulation and estimation of partial regression coefficients

Classical decomposition of a generalized additive time series model is utilized to split IT into 4 different components, comprising of constant and varying parts (Eq. 1). C_*i*_ denotes the long-term mean SST over grid box *i*, which is constant for the reconstruction period, and A_*m,i*_ represents the seasonal cycle which is regular and predictable change per month *m*, that recur every calendar year, to complete the constant part. The varying part of the model consists of T_*y,i*_ and ${{\rm{I}}}_{{m}_{y},i}$, with the former showing annual mean anomaly, while the latter shows monthly changes with respect to years termed intra-annual variability. Following the decomposition of IT, ${{\rm{I}}}_{{m}_{y},i}$ will average to zero for each grid box. This is imperative since the desired annual mean is preserved in N_*y,i*_.

1 $$HadISS{T}_{y,m,i}={C}_{i}+{A}_{m,i}+{T}_{y,i}+{I}_{{m}_{y},i}$$

**Fig. 3:**
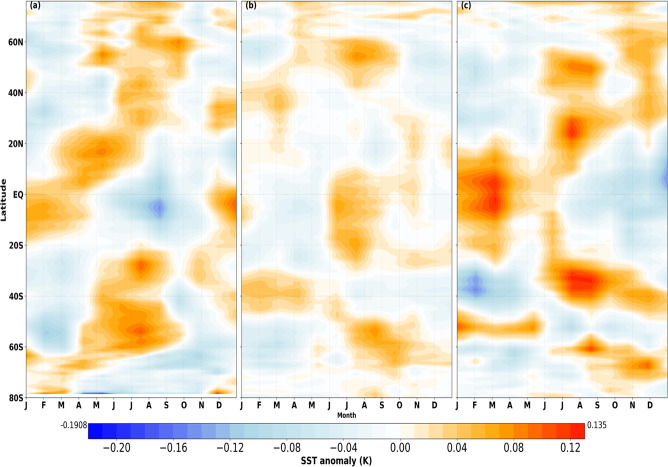
Zonally averaged partial regression coefficients showing latitudinal variations of SST anomaly (K) for all calendar months. Shown are (**a**) Nino3.4, which shows seasonal phase-locking of ENSO in the Tropical region; (**b**) DMI, which develops relative to the annual cycle during the boreal summer month (JJA) and peaks during fall (SON); and (**c**) TASI, showing the signature of seasonality with warm and cold peaks from January to June and July to December, respectively.

The aforementioned climatic indices are not independent of each other with empirical orthogonal analysis showing coupled effects of ENSO with other modes of variability (See Fig. 2a). In this case, it is also almost impossible to separate these effects if we apply the widely used Principal Component Regression (PCR) to reduces the dimensionality and then regress ${{\rm{I}}}_{{m}_{y},i}$ on the loadings. Here, we employ Partial Regression (PR) with partitioned variance using the Frisch-Waugh-Lovell Theorem FWLT^30,31^. FWLT is commonly used in the field of econometrics and it provides an alternative to the direct application of ordinary least squares by projecting linear regression models in terms of orthogonal components, which are supervised by the predictand. It is similar to PCR, but with the advantage that regression coefficients of any variable can be obtained by first partialling-out the effect of other variables in the regression model. In the least square sense, Nino3.4 exerts great influence on the spatial pattern of regression coefficients estimated for DMI and TASI, showing cold/warm tongue in the Tropical Pacific for June/December. Meanwhile, the application of FWLT controls the dominant influence of ENSO and provides distinct spatial patterns, associated with different indices (see Fig. 3).

We developed deterministic linear regression models following FWLT, which allows ${{\rm{I}}}_{{m}_{y},i}$ to vary with the Nino3.4, DMI, and TASI (Predictor Variables (PVs));

2 $${I}_{{m}_{y},i}=F(PVs)$$

therefore, the stochastic part of the PR is ignored by normalizing the PVs utilized in training the model to have constant mean and unit variance, hence giving coefficients that are unbiased in estimating ${{\rm{I}}}_{{m}_{y},i}$. However, predictions $\bar{I}{}_{{m}_{y},i}$ from this kind of model will have smaller variance compared to ${{\rm{I}}}_{{m}_{y},i}$, since there are variations that are not explained by either of the PVs^32^. On the contrary, linear regressions with stochastic terms provide variance influenced by the constant and not the PVs. In our approach, SSTs show signatures of variance influenced by PVs, which are important modes of low-frequency variability in the climate system. Thus ${{\rm{I}}}_{{m}_{y},i}$ is regressed on indices of the corresponding year, resulting in monthly stratified grid box linear models, such that;

3 $$\bar{I}\,{}_{{m}_{y},i}={\beta }_{1(m,i)}Nino3.{4}_{y}+{\beta }_{2(m,i)}DM{I}_{y}+{\beta }_{3(m,i)}TAS{I}_{y}$$

where *β*_1(*m,i*)_, *β*_2(*m,i*)_ and *β*_3(*m,i*)_ are monthly regression coefficients per grid point with response to annual Nino3.4, DMI and TASI, respectively.

The regression models gives a reconstruction with smaller amplitude of the intra-annual variability, with our statistical model outputs showing lower variance in space and time when compared with IT, but largely depict the correct spatial pattern. This low spatio-temporal variance is due to large differences in amplitude between the regression model outputs and IT. $\bar{I}\,{}_{{m}_{y},i}$ has a much smaller amplitude than its supposed true values, hence the need to rescale. During rescaling, it is important to achieve homogeneous mean and variance in space and time. These important properties of low-frequency SST boundary condition will not only provide stability in AGCM simulations, but it will also allow quantifying the relative magnitude of variability caused by changes in external forcing such as volcanic eruption, insolation or the level of increase in greenhouse gases. Therefore, we define grid-based monthly Scaling Factors (SF) as the square-root of standard deviation ratio^33^ calculated over a particular grid-box between ${{\rm{I}}}_{{m}_{y},i}$ and $\bar{I}\,{}_{{m}_{y},i}$ (see Eq. 5).

4 $$S{F}_{m}=\sqrt{\frac{stdev\left({I}_{{m}_{y},i}\right)}{stdev\left(\bar{I}\,{}_{{m}_{y},i}\right)}}$$

We calculate SF for each calendar month, and then multiply it with $\bar{I}\,{}_{{m}_{y},i}$ of the corresponding month to give ${\bar{I}}_{{m}_{y},i}^{SF}$, as this ensures a better spread around its mean values. N_*y,i*_ and the resulting ${\bar{I}}_{{m}_{y},i}^{SF}$ is then added to the constant components of HadISST_*y,m,i*_ to form a new dataset (Eq. 5).

5 $$PaleoSS{T}_{y,m,i}={C}_{i}+{A}_{m,i}+{N}_{y,i}+{\bar{I}}_{{m}_{y},i}^{SF}$$

#### Data assimilation

The application of paleoclimate data assimilation techniques to estimate past climatic variability has become more widespread in recent years. This technique involves the initial state estimate of a variable, which is then optimally combined with observations to get the best estimate of the variable. Although AGCM simulations usually provide the initial state estimate, it could result from an expansive range of different sources. In the next step of our reconstruction procedure, we use a data assimilation approach that combines our reconstructions described in the previous section with historical marine observations. These observations provide a new source of information from the 1780s that is not present in the annual mean reconstructions. However, it is spatially and temporally heterogeneous, and incorporating it is therefore not straightforward.

Although this is generally a time consuming and computationally demanding practice, there are different methods of assimilating observations into model outputs. For this study, we utilize an offline data assimilation technique called the Ensemble Kalman Fitting (EKF)^9^ as it is relatively straightforward and easy to implement.

The reconstructed SST provides an initial state estimate which is updated when observation becomes available and also using error estimates in both the observations and the initial state. Following the update procedure of Whittaker and Hamill (2012)^34^, the ensemble mean ($\bar{x}$) and the deviations from the ensemble mean (${{\boldsymbol{x}}}_{i}^{{\prime} }={{\boldsymbol{x}}}_{i}-\bar{{\boldsymbol{x}}}$) are treated separately;

6 $${\bar{{\boldsymbol{x}}}}^{a}={\bar{{\boldsymbol{x}}}}^{b}+{\bf{K}}\left(y-{\bf{H}}{\bar{x}}^{b}\right)$$

7 $${{\boldsymbol{x}}}^{{\prime} a}={{\boldsymbol{x}}}^{{\prime} b}+\widetilde{{\bf{K}}}\left({\bf{H}}{x}^{{\prime} b}\right)$$

where ***x***^*a*^ and ***x***^*b*^ denotes the analysis and the background state vectors, respectively. In our case, ***x***^*b*^ is the reconstructed monthly SST fields, ***y*** denotes the set of observations, and **H** is the forward operator, which connects the model and observational states. **K** and $\widetilde{{\bf{K}}}$, the Kalman gain matrix and the reduced Kalman gain matrix are calculated as:

8 $${\bf{K}}={{\bf{P}}}^{{\rm{b}}}{{\bf{H}}}^{{\rm{T}}}{\left({{\bf{HP}}}^{{\rm{b}}}{{\bf{H}}}^{{\rm{T}}}+{\bf{R}}\right)}^{-{\rm{1}}}$$

9 $$\widetilde{{\bf{K}}}={{\bf{P}}}^{{\rm{b}}}{{\bf{H}}}^{{\rm{T}}}{\left(({\left((\sqrt{{{\bf{HP}}}^{{\rm{b}}}{{\bf{H}}}^{{\rm{T}}}+{\bf{R}}})\right)}^{-{\rm{1}}})\right)}^{T}\times {\left((\sqrt{{{\bf{HP}}}^{{\rm{b}}}{{\bf{H}}}^{{\rm{T}}}+{\bf{R}}}+\sqrt{{\bf{R}}})\right)}^{-{\rm{1}}}$$

The Kalman gain matrix determines the weights given to the model and the observations based on their error estimates (**P**^**b**^ and **R**), as well as performs the incorporation of the observation into the model. The error in the background state, **P**^**b**^ is estimated from perturbations of the 50 analog realizations;

10 $${{\bf{P}}}^{{\rm{b}}}=\frac{{\rm{1}}}{n-{\rm{1}}}{\rm{(}}{{\boldsymbol{x}}}_{{\rm{1}}}^{{\rm{b}}}{\rm{-}}{\bar{{\boldsymbol{x}}}}^{{\rm{b}}}{\rm{)(}}{{\boldsymbol{x}}}_{{\rm{1}}}^{{\rm{b}}}{\rm{-}}{\bar{{\boldsymbol{x}}}}^{{\rm{b}}}{{\rm{)}}}^{{\rm{T}}}+.....+{\rm{(}}{{\boldsymbol{x}}}_{{\rm{n}}}^{{\rm{b}}}{\rm{-}}{\bar{{\boldsymbol{x}}}}^{{\rm{b}}}{\rm{)(}}{{\boldsymbol{x}}}_{{\rm{n}}}^{{\rm{b}}}{\rm{-}}{\bar{{\boldsymbol{x}}}}^{{\rm{b}}}{{\rm{)}}}^{{\rm{T}}}$$

where *n* is the ensemble size.

In our assimilation scheme, the estimation of measurement uncertainty for SST is adopted from Kennedy *et al*.^35^, where error estimates are decomposed into random and systematic parts, giving a range of 0.56–0.81 K and 0.37–0.8 K, respectively. Since our assimilation period (1780–1849) covers an earlier timeslice, and accompanied with even higher uncertainties, we set both error components to 1 K each, and the random part is then scaled with the square root of the number of observation, which vary spatially as provided for the gridded SST product, hence observation error estimates also vary spatially between 1 and 2 K. For a single NMAT record, the error is set to a constant value of 2 K whereas if more than one observation is available within a particular grid, the grid average is assimilated and the error is also scaled with the number of observation available within that grid. Furthermore, the observations errors are assumed to be uncorrelated, implying a diagonal observation-error covariance matrix (**R**). Therefore observations can be assimilated one-by-one. Observations are combined in a 6-month assimilation window (October–March, and April–September)^36^ and applying time localization so that monthly observational data are restricted to update on monthly basis from the half-yearly state vector. To be consistent with our assimilation window, the gridded NMAT is filtered using a 6-months running average from October–September. We also implement distance-dependent spatial localization to emit spurious long-range correlation. The length-scale parameter of the Gaussian localization function is set to 1500 km, based on the spatial decorrelation between SST anomalies at a reference point and different distances and directions. This is calculated using a spatially complete HadISST2.0 as well as the annual mean reconstruction. In both cases, these correlations does not change so much along longitudes compared to latitudes. At 1500 km, the correlation along latitude is approximately 0.65. The **H** forward operator simply extracts the value of the closest grid cell from the observation location.

Before assimilation, the observations were screened using the criteria utilized in the Twentieth Century Reanalysis (version 3, 20CRv3)^37^, with slight modification (Eq. 11).

11 $$\left|y-{x}_{b}\right| > 3\sqrt{{\sigma }_{y}^{2}+{\sigma }_{b}^{2}}$$

where ${\sigma }_{y}^{2}$ and ${\sigma }_{b}^{2}$ are the respective observation and the model error variance estimates at the closest grid cell to the observation location. The observations are assimilated in two phases. In the first, we assimilate SSTs anomalies (climatological monthly averages for 1830–1879 were subtracted from both observations and the reconstructed SST ensemble), for the analyses period 1801–1879. In the second phase, we assimilate NMAT using the regression-based reconstructed SSTs from 1780 to 1800 and the analyses obtained after assimilating ICOADS SSTs in the first phase (1801–1879).

#### Historical sea ice reconstruction via Analog Resampling Method

Many historical AGCM simulations approach the representation of sea ice concentration in a simple way, which involves the specification of modern-day monthly or seasonal climatological means. This approach has been shown to result in large biases in Arctic temperature and anomalous wind speed along sea ice edges^38^. Our reconstruction attempts to reduce these inconsistences by providing SIC that fits the spatial pattern of the reconstructed SST, and physically consistent for use in AGCM simulations. Similarly, paleoclimate reconstruction technique such as data assimilation requires a prior state estimate, and historical observations which are both not available. Although, there are several sea ice proxies, there a very few of them with high temporal resolutions^39^. Since similar SST conditions around the poles are likely to influence SIC in its neighboring region, we reconstruct SIC using the analog resampling method^10,40,41^.

**Fig. 4:**
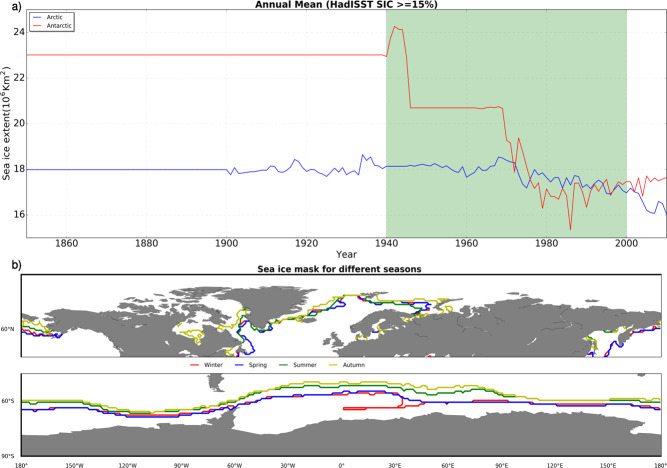
Pooling and selection of best sea ice analogs. (**a**) The annual mean sea ice extent as estimated from HadISST SIC for the Arctic (blue) and Antarctic (red). The green shaded area shows the timestamp where analogs are pooled on a seasonal basis, while (**b**) show regions masked (polewards from different lines) before selecting the best sea ice analog for both Northern and Southern hemispheres.

This method requires a pool of possible analogs, from which SIC can be drawn to fill-in for missing historical observations based on a measure of similarity between our reconstructed SSTs and its IT, which is available with SIC from 1850 onwards. However, HadISST SIC uses monthly climatological averages from 1850–1940, with no variability within this timeslice (Fig. 4a). Hence limiting the number of possible analogs on a monthly basis. Therefore, the pooling of analogs is carried-out on a seasonal basis namely; winter (DJF), spring (MAM), summer (JJA), and autumn (SON) for SIC in the second half of the 21st century (1941–2000). Furthermore, we separate Arctic and Antarctic SIC to allow inter-hemispheric variations in our reconstruction. This implies that, for each month within a season, there are 180 possible analogs for each hemisphere.

In the climatological period of HadISST SIC, sea ice reaches its maximum extent in a specific month within each season for both Northern and Southern hemispheres namely; February, March, June, and November for winter, spring, summer, and autumn, respectively in the Arctic, while the respective months in the Antarctic are December, May, August, and September (Fig. 4b). These months of maximum extents are used to delineate our region of comparison along each pole so that SSTs used in the selection of SIC analogs are in regions that are not covered by sea ice. This imposes a strict measure in comparison by neglecting sea ice regions, which are generally too cold and with a high likelihood of similarity. The best analogs are selected based on correlation coefficients between reconstructed SSTs and IT from 45° N/S to the points of maximum ice extents in the Arctic and Antarctic (Fig. 4b), respectively. Correlation measures the degree of similarity of SST patterns, and does not penalise two fields that may differ by large constant value^10^. This is plausible for the preindustrial era which has little or no trend. Using other measure of similarity such as RMSE, will penalise the fields by large constant value which is predominant in the period where the analog are pooled, hence selection of fewer best analogs. It is important to note that HadISST Arctic SIC is adopted from the Walsh dataset^42,43^ for 1901–1978 as the underlying data source. The Walsh dataset provides data for only April to August before 1953. However, HadISST applied some modifications by defining typical SIC climatology for every calendar month based on bias-corrected passive microwave data 1979–1996. The calculated climatology ensures that the general characteristics of HadISST SIC are consistent through time and adds more spatial variability. In some areas, the Walsh dataset had 100% SIC while the corrected passive microwave climatology had values ≥90%^2^. In this case, the calculated climatological values were adopted and used in the HadISST dataset. Although we pooled our SIC analog from the year 1941 to 2000 (Fig. 4a), where the added variability is evident, this constraint could hamper the seasonal SIC variability for spring and summer. The ≥90% SIC climatological values help avoid making drastic changes to the Walsh dataset in areas where the historical SIC may represent its true state.

## Data Records

An overview of the data files used in our reconstruction showing the coverage, resolution, formats, and links to the repositories where they are stored, is shown in Table 1. The resulting 50-member ensemble gridded SST and SIC datasets, in NetCDF format, can be found in figshare repository, integrated with this data descriptor^44^. We provide these datasets in two categories. To distinguish between these datasets, we adapt two naming conventions. First is the linearly regressed SST from 1000 to 1780, combined with data assimilation output spanning the period 1780–1849. These datasets named PaleoSST realization 0XX, where XX indicate the ensemble number ranging from 01 to 50. Each realization contains a netCDF PaleoSST_SIC_1000–1849_R0[01–50].nc. Secondly, we also provide the background used in the data assimilation scheme covering the period 1780–1849. It is named PaleoSST_LR_1780-1849.tar.gz and contains 50 netCDF files PaleoSST_LR_1780-1849_R0[01–50].nc.

## Technical Validation

The SST dataset is evaluated in four steps. Firstly, we assess some statistical measures of reconstruction accuracy based on IT in an overlapping period, using a single member that is randomly drawn from the 50-member ensemble. Our comparison is carried out for the validation period (1850–1879) with anomalies calculated based on a 30-year SST climatology estimated from IT (1961–1990), before and after observations have been assimilated. For this, we compute grid-based correlation coefficients (−1 ≤ *r* ≤ 1) and Root Mean Square Error (RMSE), to show the linear relationship and magnitude of error between our reconstruction and IT, respectively. RMSE is calculated between reconstruction R_*i*_ and it’s instrumental target IT_*i*_, over 30 (n) year period for all gridboxes (i) (Eq. 12). This evaluation measure is sensitive to larger errors, and as such low RMSE indicates better agreements.

12 $$\begin{array}{l}RMSE=\sqrt{\frac{1}{n}\mathop{\sum }\limits_{i=1}^{n}{\left({R}_{i}-I{T}_{i}\right)}^{2}}\end{array}$$

Furthermore, we compare intra-annual variability between our reconstruction and IT, by showing the ratio of standard deviation (IT/PaleoSST) between both datasets. This measure of comparison shows how the reconstruction spreads out from its mean value per grid-point with respect to IT. A variance ratio of 1 represents the equal magnitude of variability explained by both datasets, while values less than 1 depicts more variance in PaleoSST than IT and vice-versa. Mean Squared Error Skill Score (MSESS) is used to evaluate our reconstruction skill before and after assimilation, with respect to IT (Eq. 13).

13 $$MSESS=1-\left(\frac{\mathop{\sum }\limits_{i=1}^{n}{\left({R}_{i}-I{T}_{i}\right)}^{2}}{\mathop{\sum }\limits_{i=1}^{n}{\left(\bar{O}-I{T}_{i}\right)}^{2}}\right)$$

MSESS (−∞ ≤ *MSESS* ≤ 1) shows improvement or decline in the accuracy of the reconstruction R_*i*_ over a mean climatological period $\bar{O}$ (1961–1990), with reference to the reconstruction’s instrumental target IT_*i*_. MSESS value of 1 indicates a perfect score, while 0 shows equal skills between the reconstruction and climatology. Furthermore, MSESS values below zero denote a decline in skill compared to climatology. However this measure of skill score punishes variance; i.e. a reconstruction that has the correct variance but no skill will have an MSESS value of -1^41^. In the second subsection, we analyse the ensemble mean of the skill score metrics defined above. For this, we show the annual (Jan–Dec) and seasonal (DJF and JJA) spatial evaluation as well as the summary over all grid boxes inferred from r, RMSE and MSESS. We compute the temporal spread over typical regions of the ocean, for the whole assimilation period (1781–1879). This shows the evolution of the ensemble spread over time in different regions, before and after observations have been assimilated. The ensemble spread (EnsSpread) is a measure of the uncertainty covered by the reconstruction and thus calculated as the extention from the minimum to maximum values of all the 50-members. Lastly, we compare our SST reconstruction with other SST data sets that extend beyond 1850 namely; SODAsi 3.0 and a single member SST^4^ utilized for the reanalysis product EKF400^9,11^. For these, we calculate anomalies for 30 years (1820–1849) and compare average intra-annual standard deviations based on a reference climatology from IT (1961–1990). However, IT and SODAsi 3.0 also use ICOADS observation and are therefore not independent.

Due to the lack of monthly sea ice observations and skillful existing SIC datasets covering our reconstruction period, validation of the SIC is almost impossible. For this, we show the inter-annual variability realized in the Arctic and Antarctic from analog re-sampling of HadISST SIC, and also how the reconstruction evolves for a particular month throughout the reconstruction period.

### Evaluation of a single-member

**Fig. 5:**
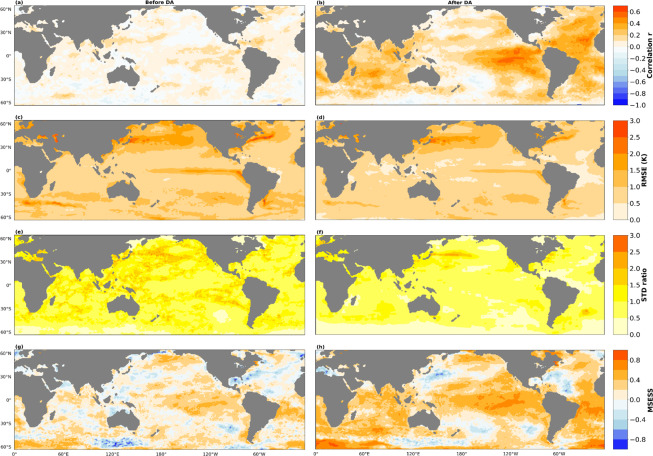
Comparison between PaleoSST and HadISST in an overlapping period (1850–1879). (**a**) Spatial correlation between both data sets before the implementing Data Assimilation (DA) scheme, while (**b**) shows the same but after DA. (**c**) and (**d**) shows the spatial distribution of the root mean square error (K) before and after DA, respectively. (**e**) The ratio of standard deviation averaged over all calendar months between PaleoSST and IT before DA while (**f**) shows the same but after DA. Lastly, (**g** and **f**) show the MSESS before and after DA, respectively.

In general, the implementation of DA improves the agreement of our reconstruction with IT as the spatial correlation coefficient increased from a maximum of 0.22 before DA to 0.61 after DA (Fig. 5a,b). The increased correlation after DA is noticeable across all basins but very pronounced in both the Atlantic and Indian sectors. The Northern and Southern extra-tropics in the Pacific also witnessed increased correlation but on a relatively smaller magnitude compared to the tropical Pacific where the correlation increased from 0.1 to 0.6, while r increased from 0.02 to 0.3 on the global scale before and after DA, respectively. The correct spatial pattern of global SST variability is recovered from the reconstruction before DA, but in regions of strong SST gradients such as the Gulf Stream, the Alghulas, and the Kuroshio-Oyashio region, the linear regression tends to produce weaker gradients. These led to high Root Mean Square Error (RMSE) in the regions before DA, and it is reduced significantly after DA (Fig. 5c,d). RMSE value over these regions decreased from 2.8 to 2.3 while along the coast of Ecuador, the value improve from 2.3 to 1.8, before and after DA, respectively. Furthermore, RMSE is reduced in the Equatorial region with the Atlantic basin showing the most pronounced improvement with values dropping from 1.5 before DA to 0.3 after DA. On the global average, our SST reconstruction gives a better agreement with IT after DA with RMSE of 0.74 compared to 0.92 before DA. The multiproxy temperature reconstructions utilized here provide a good representation of inter-annual and decadal SST variability, while the sub-grid variability on intra-annual timescale is empirically scaled with reference to IT. The result shows a good agreement between our reconstruction and IT, with a variance ratio less than or equals to 1 in most of the grid points, before and after DA (Fig. 5e,f). Our regression approach also shows good skill in reconstructing sub-grid scale SST variability with positive MSESS in many grid points, particularly over regions where climatic indices used in training the regression models are estimated. However, the implementation of DA improves the reconstruction skill over the entire ocean basins, except along the Gulf of Mexico, the eastern part of the Kuroshio-Oyashio region, and in most of the Southern Pacific (Fig. 5g,h). The MSESS increased from an average of 0.37 to 0.59 and 0.20 to 0.42 over the Equatorial Pacific and in the Atlantic sector of the Equatorial region, respectively. Negative MSESS are evident in the North Atlantic before DA, and improved to an average of 0.39 after DA. Furthermore in the Southern Ocean, negative MSESS is pronounced south of Australia between 110° E–160° E and 50° S–60° S, and persists even after DA, but with reduced magnitude. The change in MSESS from negative to positive over the Hudson Bay is also evident, while the average along the Bering Strait increased from 0.14 to 0.36, before and after DA, respectively. In our alternative experiment, the correlation coefficient increased from a maximum of 0.22 in the initial to 0.34 (Figure S1a and b). Increased correlation is evident in the Southern Ocean, North Atlantic, and Indian Ocean. In the ENSO region, the correlation decreased in the alternative experiment. The RMSE increased globally in the alternative experiment, especially in those regions of strong SST gradients such as the Gulf Stream, the Alghulas, and the Kuroshio-Oyashio region (Figure S1c and d). This increased RMSE implies that training the model outside the satellite era gives regression coefficients that produce weaker gradients than the initial experiment. In both cases, the linear regression shows good skills in reconstructing sub-grid scale variability with positive MSESS evident over many grid points (Figure S1e and f), but with patches of negative MSESS seen over the North Atlantic, South Australia, and North Pacific. We conclude that training the linear regression in the period where the target dataset is reliable (such as in the satellite era, in this case) will ensure that the regression coefficients gives the best obtainable, as seen in the reduced RMSE values in the regions of strong SST gradients (Figure S1c and d).

### Skill score metrics ensemble mean

**Fig. 6:**
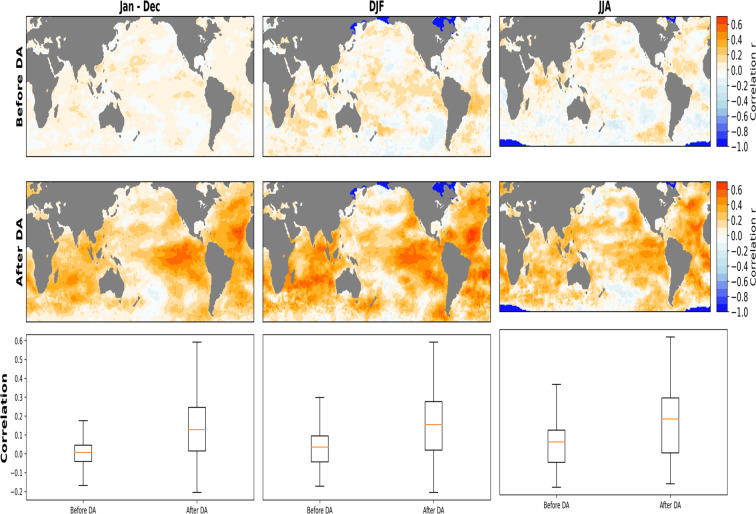
Spatial correlation between the ensemble mean PaleoSST and HadISST in an overlapping period (1850–1879). The first row shows the correlation before implementing Data Assimilation (DA) on Annual (Jan–Dec), boreal winter DJF and boreal summer JJA timescales. The middle row shows the correlation after DA on annual and seasonal timescales (DJF and JJA). The last row summarizes the annual and seasonal spatial correlation over the same overlapping period.

**Fig. 7:**
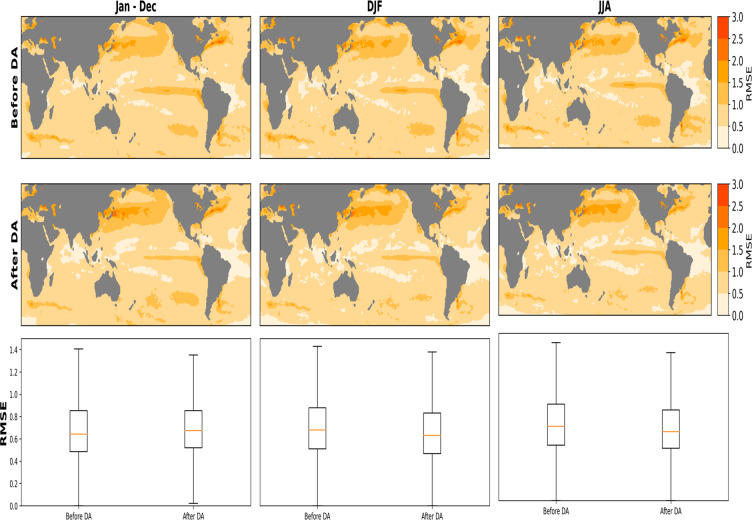
Root Mean Squared Error (RMSE) ensemble mean between PaleoSST and HadISST in an overlapping period (1850–1879). The first row shows the RMSE before Data Assimilation (DA) scheme, on Annual (Jan–Dec), boreal winter DJF and boreal summer JJA timescales. The middle row shows the RMSE after DA on annual and seasonal timescales (DJF and JJA). The last row summarizes the annual and seasonal RMSE over the same overlapping period.

The ensemble means of the correlation between all 50-members and IT before DA shows its best agreement in boreal winter compared to summer and on annual timeslices (Fig. 6). Correlation values are relatively high in the tropical region with a maximum of 0.31 during winter and 0.22 on the annual timescale, while during summer, a correlation value of 0.33 is evident in the Indian Ocean. The implementation of DA improves the correlation for all considered seasons and also annually (Fig. 6). Similarly, the highest correlation after DA is noticeable in winter, where the values reach 0.6 in the tropical regions. Generally, positive correlations are evident over the Atlantic and Indian basins in all the seasons considered, while the southern and northern extratropics in the Pacific witness a mix of positive and negative correlations. In winter and summer, sea ice edges show a correlation of -1. These negative correlations are due to the lack of variability in SST over these regions in the IT. RMSE between the 50-member and IT shows its most notable differences in the Tropical Atlantic, where the error reduced from an average of 0.9 before DA to 0.12 after DA (Fig. 7). RMSE in Tropical Pacific and especially in regions of cold/warm tongue induced by ENSO is reduced in its mean value and spatial extent after DA. The pattern of RMSE appears to be the same for all considered seasons and on the annual timescale. In the RMSE mean, regions of strong SST gradients such as the Gulf Stream, the Alghulas, and the Kuroshio-Oyashio region showing weaker SST gradient while evaluating a single member show no appreciable difference before and after DA. These contrasting patterns in single-member and the ensemble mean indicates variations between the ensemble members. It also gives an indication that a wide range of spatial uncertainty is covered within the sample space of the reconstruction. The summary boxplots show reduced RMSE on seasonal (DJF and JJA) and annual basis.

**Fig. 8:**
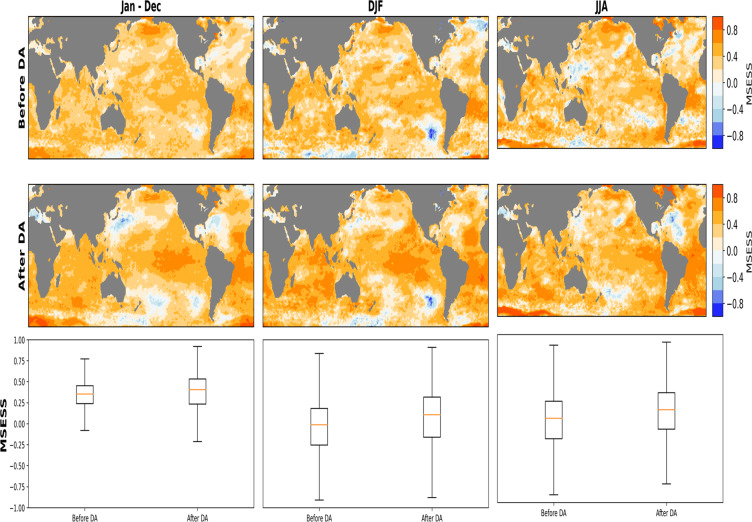
Mean Squared Error Skill Score (MSESS) ensemble mean between PaleoSST and HadISST in an overlapping period (1850–1879). The first row shows the MSESS before Data Assimilation (DA) scheme, on Annual (Jan–Dec), boreal winter DJF and boreal summer JJA timescales. The middle row shows the MSESS after DA on annual and seasonal timescales (DJF and JJA). The last row summarizes the annual and seasonal MSESS over the same overlapping period.

Lastly, we evaluate the ensemble mean of MSESS considering all 50-members and IT. In general, positive MSESS is evident for all seasons, before and after DA but with a few patches of negative values (Fig. 8). During winter, negative MSESS is more pronounced along the coast of Chile. These negative MSESS persists even after DA. The same is the case for the annual timeslice but on a relatively small magnitude. Similar to the single ensemble member considered, the negative MSESS evident south of Australia between 110° E–160° E and 50° S–60° S is also visible. The summary boxplot indicate a better skill on the annual scale with respect to seasonal skills, with the average MSESS increasing from 0.30 to 0.36. Also on the annual scale, a maximum MSESS of 0.85 is evident After DA, which is an improvement from before DA with a corresponding value of 0.76. The summary over DJF and JJA are quite similar show little improvement in MSESS before DA to after DA, in terms of the mean and maximum values. In contrast to the annual MSESS, the seasonal skill score have higher magnitude of negative values that is evident in the Southern Ocean and the North Atlantic.

### Ensemble spread as reconstruction uncertainty

**Fig. 9:**
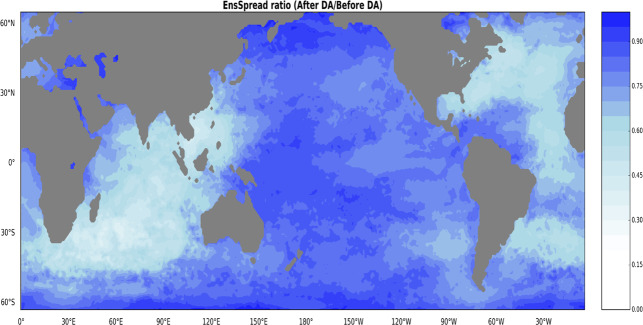
Ensemble spread (EnsSpread) ratio between reconstructed SSTs before and after data assimilation (After DA/Before DA) calculated over the whole assimilation period (1781–1879).

**Fig. 10:**
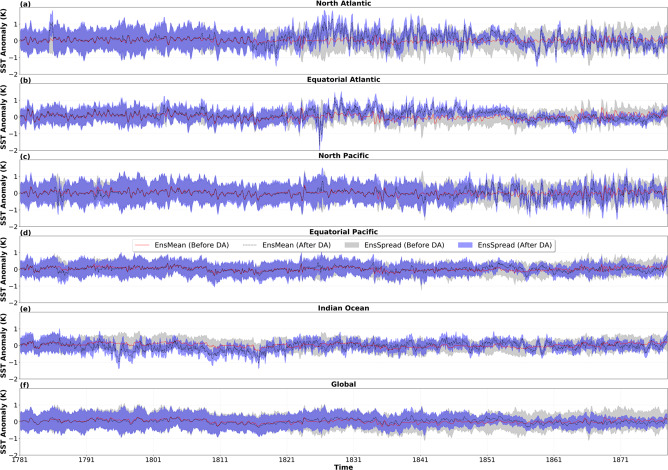
The temporal evolution of ensemble spread (EnsSpread) before and after DA, calculated as mean anomalies over the entire assimilation period (1781–1879) for selected ocean regions. Shown are (**a**) North Atlantic (291°–358°, 21.5°–65.5° N) (**b**) Equatorial Atlantic (291°–358°, −22.5–21.5° N) (**c**) North Pacific (120°–238°, 21.5°–55.5° N) (**d**) Equatorial Pacific (120°–238°, −22.5°–21.5° N) (**e**) Indian Ocean (34.5°–108°, −22.5°–14.5° N) and (**f**) Global. The grey shade and red line show the EnsSpread and ensemble mean (EnsMean) before DA, while the blue shade and black dashed line depicts the EnsSpread and EnsMean after DA, respectively.

The ensemble spread (EnsSpread) provides a measure of the range of uncertainty covered by the reconstruction. Generally, the spatial and temporal spread of the ensemble SST reconstruction witness reductions after DA. These reductions are more pronounced in the Atlantic and Indian basins (Fig. 9). However, in the Pacific and the Southern Ocean, the variance ratio is reduced but on a relatively lower magnitude. This high variance ratio in the Pacific and the Southern Ocean is due to the lower number of observations in these basins for most of the assimilation period. The temporal evolution of EnsSpread also shows that DA impacts differently across typical ocean basins. Spread is significantly reduced in the Atlantic (Fig. 10a,b) and Indian (Fig. 10e) basins in most of the assimilation period while EnsSpread remains relatively constant over the Pacific (Fig. 10c,d) due to low coverage of historical marine observations over the basin.

Furthermore, Ensemble Mean (EnsMean) across all considered regions show a smaller amplitude of variability before DA (Fig. 10). Increased variability is most pronounced in the Indian and Atlantic basins, where the temporal signature of multidecadal variability becomes more evident in the North Atlantic, after DA. The implementation of DA also leads to an improved variability over the Pacific basin, and the changes are apparent from the mid-1840s. On the global scale, temporal EnsSpread also witnesses reductions with the increasing availability of marine observations, which are evident from the early part of the 19th century (Fig. 10f).

### Comparison with available historical monthly SST data sets

**Fig. 11:**
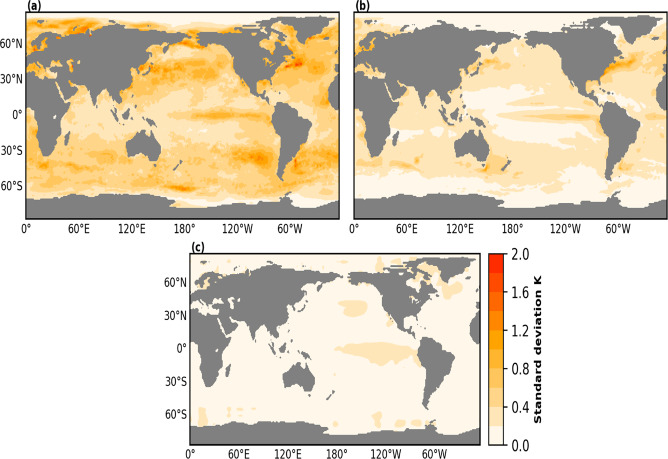
Average intra-annual standard deviation (K). (**a**) PaleoSST, (**b**) SODAsi3.0 SST and (**c**) augmented version^9,11^ of Mann *et al*., (2009) SST, in an overlapping period (1820–1849).

We compare the intra-annual variance of our reconstruction with existing global monthly SST data sets. SST from sparse observational input SODAsi3.0 and the augmented version^9,11^ of a multiproxy reconstructed temperature described in Mann *et al*. (2009)^4^ are utilized in this respect. The result shows a similar spatial pattern of variability between our reconstruction (Fig. 11a) and SODAsi3.0 SST (Fig. 11b), with an overall high variability at high latitudes compared to the tropics. In general, the magnitude of variability is more pronounced in our reconstruction. Both data sets show that Oceanic variability reaches its highest value along the Gulf Stream in the North Atlantic and the Kuroshio region in the North Pacific. The high intra-annual variability in our SST reconstruction results from the significantly large number of historical marine observations assimilated into it, compared to sparse input utilized for SODAsi3.0.

The magnitude of intra-annual variability in the augmented version of Mann *et al*. (2009) SST is significantly low across all grid points (Fig. 11c). In contrast to other data sets that make use of observational inputs, this dataset utilized only information retrieved from paleoclimate proxies. It is also decadally smoothened hence it has low variability on the intra-annual timescale.

### Variability in SIC reconstruction

**Fig. 12:**
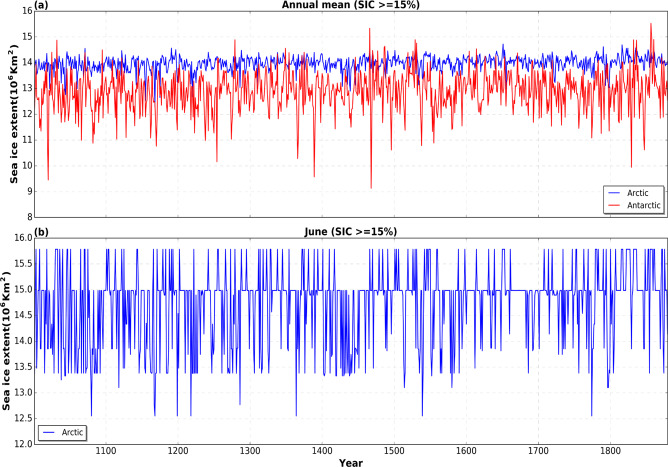
Variability in reconstructed sea ice concentration. (**a**) Annual mean sea ice extent (1000–1849), showing inter-annual variability in a single ensemble member for the Arctic (blue) and Antarctic (red). (**b**) Arctic sea ice extent for a specific month (June) across the reconstruction period.

Since sea ice is reconstructed to fit the different realization of PaleoSST, we evaluate sea ice in terms of its variability over time. The analog resampling of HadISST SIC proves to be more effective in reconstructing annual than monthly mean sea ice extents for Arctic and Antarctic (Fig. 12a), although with more variance in the Antarctic. On the contrary, it dampens month-to-month variability due to the limited number of possible analogs, as well as intra-seasonal variability as a result of seasonal pooling. For a specific month across the whole reconstruction period, the measure of similarity utilized in selecting the best analogs only picks few analogs (Fig. 12b). Similarly, there is a likelihood that months in the same season within a year will have the same SIC in the Arctic and or the Antarctic.

## Usage Notes

This dataset is suitable for monthly historical AGCM simulations, since our reconstruction targets monthly mean values. It is important to note that most climate models utilize these values as representatives of mid-months, thereby obtaining daily estimates via linear interpolation between months. Therefore, specifying these monthly means in climate models will not conserve the values, it dampens seasonal, intra-annual, and inter-annual variability^45,46^. To correct this constraint, higher values which will average back to the monthly mean in the GCM simulations must be specified. There are many algorithms to overcome this constraint^46–48^, but the most widely used method is prescribed for the second phase of the Atmospheric Model Inter-comparison Project (AMIP II). We strongly recommend that the AMIP II interpolation scheme^46^ is applied before simulations are implemented with this dataset, as this scheme has been used within a wide range of modeling communities and have shown consistent results within the framework of AMIP and beyond^49^. The data set can also be used for other purposes requiring monthly, seasonal and annual SST and SIC from 1000 to 1849.

## Supplementary information

Supplementary Information

## Data Availability

Three main types of codes were used in generating the datasets. Another one is also developed to interpolate between mid-months, while still conserving the monthly mean values. For spatial regression coefficients, we utilize a shell script calculates spatial regression for different calendar months, implementing various Climate Data Operators commands, and another shell script is used to select best sea ice analogs based on correlation coefficients between reconstructed subpolar SSTs and its instrumental target are available on GitHub (https://github.com/shamakson/PaleoSST_SIC_ARM.git). The basic form of the data assimilation code written in R, is available on GitHub (https://github.com/jf256/reuse.git) and a python script that implements the AMIP II interpolation scheme is also available via GitHub (https://github.com/shamakson/AMIP_bc_interpolation.git).
