# Supplementary material for: An ensemble reconstruction of global monthly sea surface temperature and sea ice concentration 1000–1849
Source: Sci Data. 2021 Oct 4;8:261. doi: 10.1038/s41597-021-01043-1 (PMC8490424; doi:10.1038/s41597-021-01043-1)
Supplement: Supplementary file 1 — Supplementary Information [file 41597_2021_1043_MOESM1_ESM.pdf]

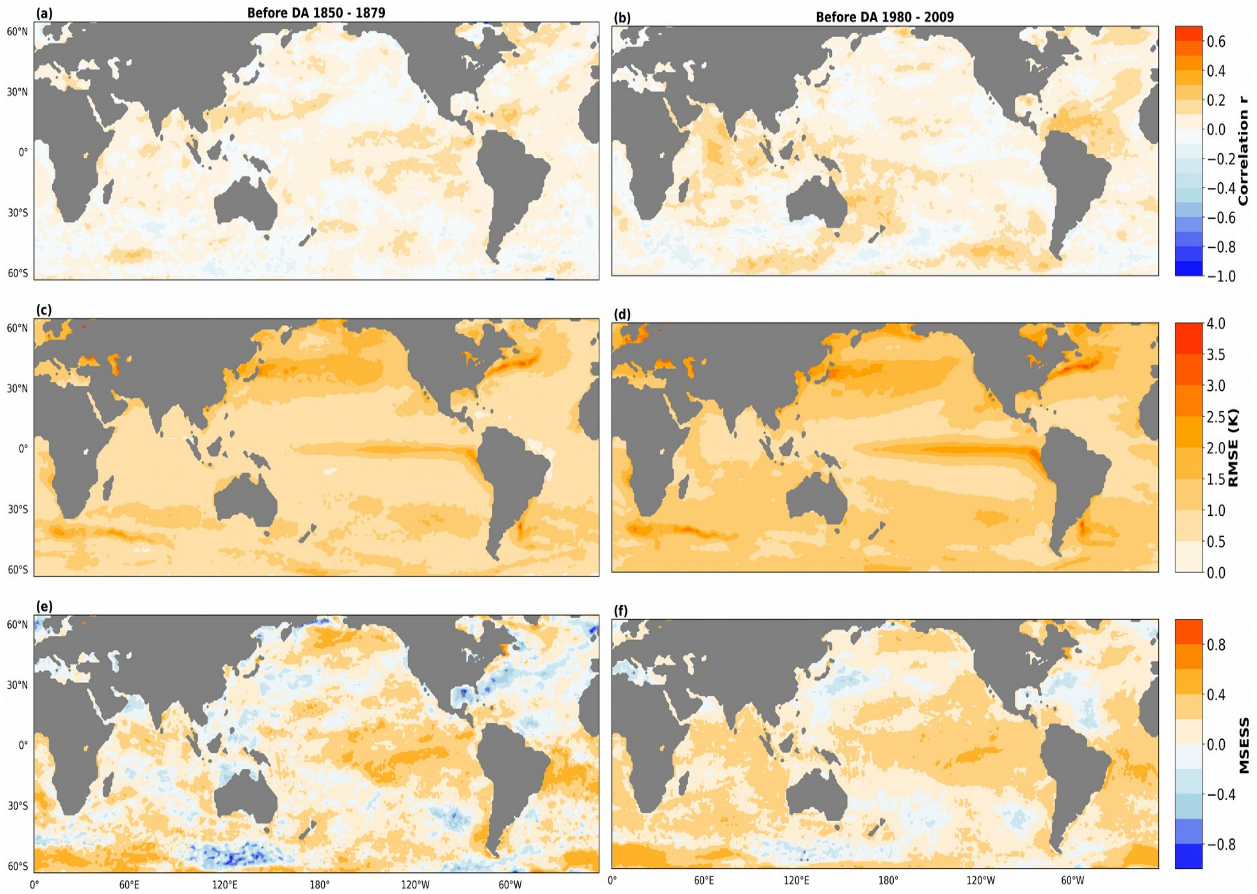

Fig. S1: Comparison between the initial (1880 – 2010 / 1850 – 1879) and alternative (1850 – 1978 / 1980 – 2009) training/validation period without data assimilation. Shown are the spatial correlation (a and b), RMSE (c and d), and MSESS (e and f) between the initial experiment and HadISST in an overlapping period, respectively.
